# First Lines of Therapeutics

**Published:** 1880-01

**Authors:** 


First Lines of Therapeutics, as Based on the Modes of the Process of Healing as occurring spontaneously in Disease; and on the Modes and the Process of Dying as Resulting naturally from Disease. In a series of Lectures by Alexander Harvey, M.A., M.D., (Edin.) Emeritus Prof. of Mat. Medica in the University of Aberdeen, formerly Lecturer therein, etc., etc. D. Appleton & Co., N. Y. 1879.

These lectures are designed to illustrate and to more clearly define the scientific bearing and relations of the two fundamental laws of therapeutics and pathology—the *vis medicatrix naturæ*, and the processes of *decay and death.* To the virtual neglect of instruction on these subjects in our college curriculum, the author ascribes much of the misconception that exists in regard to them.

In discussing the subject, the supremacy is given to nature, while art comes in for a secondary portion of the glory.

Time and space will not permit a lengthy analysis of the argument, but we will say that the book is very interesting as well as instructive.

